# Predictive multispecies constraint-based metabolic modeling: case studies and best practices

**DOI:** 10.1093/bib/bbag196

**Published:** 2026-04-30

**Authors:** Joshua A M Kaste, Megan L Matthews

**Affiliations:** Department of Civil and Environmental Engineering, Grainger College of Engineering, University of Illinois Urbana-Champaign, 205 North Mathews Ave, Urbana, IL 61801, United States; Department of Civil and Environmental Engineering, Grainger College of Engineering, University of Illinois Urbana-Champaign, 205 North Mathews Ave, Urbana, IL 61801, United States; Carl R. Woese Institute for Genomic Biology, University of Illinois Urbana-Champaign, 1206 West Gregory Drive, Urbana, IL 61801, United States

**Keywords:** flux balance analysis, constraint-based modeling, metabolic modeling, microbiome

## Abstract

The last decade has seen a proliferation of studies and techniques for the modeling and analysis of metabolic interactions between distinct species, including microbes, plants, mice, and humans. Many studies in this area are explanatory, but a subset present predictive models aimed at either achieving specific biotechnological aims or furthering our understanding of the biological systems in question. Recent work suggests that the analysis frameworks and models that have been developed in the area of microbiome research are unreliable for accurately characterizing species-to-species interactions. In light of this development, the development of this subfield of metabolic modeling and its latest stumbling block are put into historical context. Several case studies of validated and predictive multispecies constraint-based metabolic models are then discussed. From these case studies, general principles are extracted that readers and practitioners in this area may find useful for evaluating and carrying out future multispecies metabolic modeling.

## Introduction

In the last 20 years, there has been a proliferation of studies extending constraint-based modeling techniques—primarily flux balance analysis (FBA) [1] and variants—to the study of natural [2] and synthetic [3] microbial consortia and interactions between eukaryotes and microbial symbionts [4–6], which will be referred to in this review with the umbrella term “multispecies models.” The models developed in these studies have been used to optimize bioreactor setups for high-throughput biochemical synthesis [3], understand the evolution of symbiotic relationships in animals [4], characterize the costs and benefits of metabolic exchanges between species [5, 6], and explain the dynamics of pathogen proliferation in human tissues [7]. This progress in the literature has taken place alongside and made use of computational methodologies for predicting microbe-to-microbe interactions [8–13]. Previous reviews [14–16] have covered the development of this field in great depth and we encourage readers to read these references for insight into the historical development of this area.

Due to the great degree of variation in how microbial organisms interact with their hosts and with each other in communities, there is no general framework for describing and predicting the outcomes of interactions. Instead, different modeling methods have been developed that mathematically encode specific biological characteristics of the particular multispecies systems under investigation. In the well-studied example of the human microbiome, it is known that the functional outcomes of the human–microbiome symbiosis are affected by a myriad of factors, including metabolism but also host–microbe signaling, immune responses, and behavior [17]. A comprehensive mathematical model of such a system is, at present, beyond our grasp, but constraint-based metabolic modeling approaches can still extract meaningful insights by focusing on specific subsets of the underlying biology. In the human gut microbiome, for example, one might expect strong spatial differentiation in community composition [18]. For such cases, methods like BacArena have been developed that can describe such spatial heterogeneity through the use of an agent-based modeling approach [19]. In BacArena, the growth environment is represented by a grid and each cell of the grid contains a metabolic network, representing the metabolic activity of an individual organism [19]. When using such approaches, however, one must also carefully consider the many biological aspects of host–microbe or microbe–microbe interactions not captured by these models that might affect model predictions. If one used a community modeling method that predicts individual microbial growth rates based on media composition and metabolite cross-feeding [11] to describe the human gut microbiome, for example, changes in microbial abundance resulting from host immune responses to these microbes (e.g. [20]) could result in significant prediction error.

A series of recent studies have highlighted the limitations of some of the aforementioned methods and models with respect to accurately predicting microbial interactions [21, 22]. The rapid development of a family of methods in constraint-based modeling followed by disappointing reports of out-of-training sample performance has historical precedent. The mid-2000s to the mid-2010s saw a similar rise in methods meant to integrate transcriptomic and proteomic expression datasets with FBA modeling, also culminating in an unfavorable benchmarking study [23]. This spurred the development of more sophisticated omic integration methods in studies featuring more extensive and consistent validation practices inspired by [23]. Prominent studies presenting improved omic integration algorithms [24–26] were published in light of this benchmarking analysis. These new studies not only explicitly discussed the findings of [23], but used many of the same datasets and models [27–29]. These studies validated their models by comparing their estimated fluxes with fluxes generated without omic integration but using parsimonious FBA [30, 31], which, unlike standard FBA can predict unique flux distributions and allow for straightforward comparison of measured and predicted fluxes.

This temporary stumbling block was ultimately beneficial to the field as it refocused researchers’ efforts on method and model validation. It is valuable to reflect on how this setback was overcome and extract lessons and guidelines that may be useful to incorporate into multispecies modeling. To do this, we will first review what is meant by “predictive” modeling in this field and the deficiencies recently highlighted in the literature. Then, with reference to the development of omic integration methods, we discuss three overarching questions that practitioners and readers of multispecies metabolic modeling studies should consider to ensure robust interpretation or development of predictive multispecies metabolic models. Finally, we highlight a series of studies that provide clear answers to these questions and address the major challenges facing predictive multispecies modeling in order to make useful predictions.

### Predictive and explanatory multispecies modeling

Since the problems highlighted in [21, 22] are problems of predictions, it is important to clearly define what a predictive model is and what it looks like in the context of multispecies metabolic modeling. We use the distinction between predictive, explanatory, and descriptive modeling described by Galit Shmueli [32]. In this formulation, predictive models are those built for the express purpose of predicting future or novel observations upon which the original model was not trained. This can be distinguished from explanatory models, which are built to test a causal theory, or descriptive models, which simply describe data sets without making predictions or assessing causal relationships. Since metabolic modeling exercises presuppose that observed input and output data can be related through metabolic activity, strictly descriptive models do not really exist in this field. Because of this, we will focus on predictive and explanatory models. For our purposes, the clearest delineation between these approaches is the emphasis on prediction accuracy on novel data that the model was not originally trained on, in the case of predictive models, and the emphasis on model fitting and subsequent inferences about the nature of the system under investigation in explanatory modeling. More precisely, we can refer to Schmueli’s formulation [32], where they defined both predictive and explanatory models as being of the generic form:


1 \begin{eqnarray*} E\left(\boldsymbol{Y}\right)=f\left(\boldsymbol{X}\right) \end{eqnarray*}

which itself represents the experimental realization of a theoretical relationship:


2 \begin{eqnarray*} {\displaystyle \begin{array}{c}\boldsymbol{\gamma} =\phi \left(\boldsymbol{\chi} \right)\end{array}} \end{eqnarray*}

where *f* is a model that approximates a real process $\phi$, and ***X*** and ***Y*** are measured variables that represent the theorized quantities $\boldsymbol{\chi}$ and $\boldsymbol{\gamma}$, respectively. In an explanatory model, the modeler wants to assess how well the model *f* represents the real process $\phi$. They can evaluate this by comparing their modeled outputs $E\left(\boldsymbol{Y}\right)$ with the observed outputs $\boldsymbol{Y}$. To parameterize their model *f* as accurately as possible, all available observed inputs $\boldsymbol{X}$ and outputs $\boldsymbol{Y}$ are used for fitting. Hypotheses about the underlying biological or biochemical system can then be assessed based on the fitted parameters. For example, one could fit a linear model relating 10 measurable characteristics in a sample of 100 humans (height, age, sex, etc.) to their weight. After fitting, the magnitudes and statistical significance of the coefficients for each of the dependent variables might be examined to assess alternative hypotheses about which factors are most strongly correlated with height.

This contrasts with a predictive modeling exercise, where the modeler wants to demonstrate that they can make accurate predictions using *f*. This predictive accuracy can be determined by measuring new outputs (${\boldsymbol{Y}}_{\boldsymbol{novel}}$) given novel inputs (${\boldsymbol{X}}_{\boldsymbol{novel}}$) and assessing the agreement between ${\boldsymbol{Y}}_{\boldsymbol{novel}}$ and their modeled outputs $E\left({\boldsymbol{Y}}_{\boldsymbol{novel}}\right)$. Alternatively, this can be done by separating the observations $\boldsymbol{X}$ and $\boldsymbol{Y}$ into training and testing data, such that the model is fit using only ${\boldsymbol{X}}_{\boldsymbol{training}}$ and ${\boldsymbol{Y}}_{\boldsymbol{training}}$ and the model is assessed in the end by comparing the predictions $E\left({\boldsymbol{Y}}_{\boldsymbol{testing}}\right)$ to ${\boldsymbol{Y}}_{\boldsymbol{testing}}$. To contrast with the prior example, the predictive modeler, instead of fitting all the data to their linear model all at once, might fit only 50% of the data to parameterize the model and then assess it based on its ability to accurately predict the heights of the remaining 50% of the sample. Oftentimes, strong predictive models may have structures that are not easily amenable to biological/biochemical interpretation, making the hypothesis testing that is the end-goal of explanatory modeling more difficult (e.g. deep neural networks). For biologists and biochemists, explanatory models have historically been the dominant paradigm and favorable goodness-of-fit is often assumed to correspond to high predictive accuracy. However, this is not the case; the only way to ensure the predictive accuracy of a model is to assess predictive accuracy as part of the model’s parameterization/training. In the case studies below, this model fitting and evaluation nomenclature is used to clearly describe how and what is being predicted in each study.

For many systems investigated using multispecies modeling methods, the end goal envisioned by practitioners of such metabolic modeling techniques is often to use modeling to inform biotechnological modifications for chemical production or therapeutic purposes (Table 1). Predictive models are necessary for these applications because they require predicting the behavior of the systems under consideration under novel circumstances [3, 5]. Since such models appear to be a desirable end for this area of multispecies modeling, it is prudent for practitioners and readers to consider what predictive modeling in multispecies modeling entails and what unique challenges arise when conducting such studies.

**Table 1:** A summary of the types of multispecies models seen in the literature, along with example references and brief descriptions.

Category	Context	Notes	Example references
**Microbe–microbe interactions**	Pathogenic interaction	Studies in which interactions between members of a microbial community are explicitly modeled, but interaction with a host is limited (e.g. external nutrient supply is parameterized based on host)	[7]
	Commensal/mutualistic		[4, 33, 34]
	Bioreactor	Studies where the study system is an artificial microbial consortium in a bioreactor, typically intended for engineering application.	[3, 35]
**Explicit host–microbe interactions**		Studies in which metabolic models describing a host and one or more microbial species are explicitly connected and optimized together.	[5, 6]
**Implicit host–microbe interactions**		Studies in which optimization is used to predict behavior in an individual species model under conditions of interaction with another species, but without any explicit coupling and interaction between models.	[36, 37]

### What do predictive success and failure look like in multispecies modeling?

Unlike traditional single-species metabolic modeling, the purpose of multispecies modeling is to reproduce or predict metabolic interactions between organisms as well as the effect, such as a change in growth rate, on those interacting partners. As such, it stands to reason that a successful application of multispecies modeling needs to be capable of one or more of the following predictive use-cases under conditions that the model was not trained on:

1. Predict the relative abundances of species in a consortium and/or the relative growth rates of partners in a symbiotic relationship.
2. Predict the identity and quantity of metabolites exchanged between species, or exported to the growth medium, and the efficiency of these exchanges and exports.
3. Predict the changes in the individual species’ flux distributions when growing in isolation and together.
4. Predict the impacts of genetic perturbations or other biotic or abiotic perturbations on any of the quantities from (1) through (3).

These are multispecies extensions of the kinds of validations recommended for constraint-based models [38]. In contrast to single-species modeling, where empirical flux distributions derived from ^13^C-Metabolic Flux Analysis (^13^C-MFA) experiments are sometimes available for validation purposes, this is exceedingly rare in multispecies modeling. ^13^C-MFA workflows have been developed and deployed [39], but for most sets of interacting species this data will be unavailable and relatively difficult to generate, so (1), (2), and (4) from the list above represent the information that’s most likely to be applicable for most studies.

Joseph *et al.* 2024 [21] and Scott *et al.* 2023 [22] evaluate the ability of a diverse set of multispecies modeling frameworks and models to successfully achieve predictive use-cases (1) and (2), respectively. Similar to what was observed in the benchmarking study evaluating omics-integration with FBA models [23], both studies find that many of these methods and models fall short in achieving these predictive goals. As such, there is a need for closer attention to how these multispecies models are used and developed.

In [21], the authors evaluate the performance of the Microbiome Modeling Toolbox [10], MICOM [9], and COMETS [11] for predicting the outcome of pair-wise metabolic interactions between species in multispecies metabolic models. Specifically, they compute interaction strength values by comparing the predicted maximal growth rates in monoculture and co-culture [21]:


3 \begin{eqnarray*} ratio=\frac{x_{co}}{x_{mono}} \end{eqnarray*}

where *x_co_* is the growth rate of an interacting partner in co-culture and *x_mono_* is the growth rate of that same partner in monoculture. This is an example of predictive use-case (1).

The authors compare curated genome-scale metabolic models with models from the AGORA database, which is a set of 773 semi-curated genome-scale metabolic models from organisms that can be found in the human gut microbiome [40]. Across all of the tools tested and using AGORA models, the authors report poor correlation between measured and predicted monoculture growth rates (0.093–0.239) and co-culture interaction strength (−0.293–0.072) [21]. The correlation between empirical and predicted monoculture growth rates is substantially higher when the analysis is delimited to curated models simulated on media representing a Western diet, 0.759, but is similarly bad when simulating using *in vitro* media, at 0.176. However, the improvement in the monoculture predictions seen from using curated models only translates into a marginal improvement in the correlation between measured and predicted interaction strengths in co-culture. The best performing co-culture model and media composition (the COMETS method with the *in vitro* media composition) achieved a correlation of only 0.291.

The substantial reduction in predictive accuracy for monoculture growth rates when using the AGORA models raises the question of whether the quality of the GEMs themselves may play a key role in poor predictive accuracy. Indeed, problems with the AGORA models were noted shortly after their initial dissemination by Babaei and colleagues [41]; the problems they note include extremely unrealistic growth rates, extremely low growth yields, and an inability for many of the models to grow under the expected anaerobic conditions of the human gut environment, which they ought to be able to grow in. Since the original release of the AGORA models, a new resource called AGORA2 was released [42], representing 7302 different strains and 1738 different species. An independent, systematic evaluation of the AGORA2 resources has not been performed, so it is possible that the curation processes taken in constructing them would result in better overall performance.

In Scott *et al.* 2024 [22], on the other hand, the authors evaluate a series of qualitative and quantitative metrics for a series of microbial consortia modeling techniques, including constraint-based modeling tools. Addressing predictive use-case (2), they find that although some of the assessed methods [8–10, 13, 43] can make accurate predictions of individual and community growth rates in a consortium between *Clostridium autoethanogenum* and *Clostridium kluyveri*, almost all predictions of major metabolite productions fluxes are either inaccurate or extremely imprecise. These findings help contextualize the results of [21], suggesting that even in the minority of cases where a combination of models, media conditions, and optimization methods can perform adequately on interaction strength prediction, this likely does not translate into another key output of such consortia models, which is metabolite production rates.

Despite these challenges, examples of successful predictive modeling of multispecies systems exist in the literature [3, 6, 7]. Based on a review of the existing multispecies modeling literature and retrospective consideration of the omic integration literature, we have formulated a short set of questions that we believe both practitioners and consumers of multispecies modeling studies may benefit from considering. For each question, we provide our rationale for why this question deserves consideration. Following these questions, we highlight a series of studies, each of which provides clear answers to these questions and tackles, under different circumstances and with different goals, the big challenges facing predictive multispecies modeling, and succeeds in making useful predictions.

We would also like to emphasize that the specific case studies in this review were chosen because they covered a range of different organisms and consortia and featured a diversity of approaches for validating their predictions. The omission of studies focusing on animal gut microbiomes, for example, or studies using shotgun metagenomics techniques, is not meant to suggest that attempts to develop predictive models in these areas have been less successful (for a recent example of a successful predictive model of an animal gut microbiome using shotgun sequencing, see [44]).

### Guiding questions and motivations

Both before and after the publication of [23], several general themes emerged in the literature on omic integration that can also be applied to the field of multispecies modeling. First, although papers in the field did not explicitly discuss the distinction between predictive and explanatory models, skilled practitioners understood the limitations of various models and frameworks and tailored their use accordingly. The GIMME algorithm [45], for example, was constructed and validated to generate context-specific metabolic models that, when optimized, showed a high concordance between flux distributions and measured expression data, instead of generating flux predictions in good agreement with fluxes measured directly or using techniques like ^13^C-MFA. The resulting models represent a best-guess of what the flux distributions of a particular organism, tissue, or organ would look like if its fluxes mirrored patterns in expression data while also abiding by mass balance and other imposed constraints. However, the original study did not show that the resulting models, for example, produce better growth rate predictions than ones not using this algorithm. To compensate for this, studies that used the GIMME algorithm for generating a predictive model typically validated one or more predictions after using it in the model construction process. This highlights the importance of carefully considering the nature of the models and workflows used in a study, which motivates Q1 below.

Second, evaluations of algorithms began to consistently use a standardized set of extensive multi-omics datasets. A perennial challenge in metabolic modeling is that many important, predictable outputs of these models are difficult (e.g. compartmentalized metabolite concentrations) or impossible (e.g. steady state fluxes themselves) to directly measure. Indeed, this is part of the reason why metabolic modeling techniques have become necessary and widely used, and makes validation particularly difficult. Going into a predictive modeling exercise understanding the intersection between available experimental data and predictions that can be made with the predictive model is crucial. In omic integration studies for predicting internal metabolic fluxes, for example, only a select few systems have the kinds of multi-omic datasets necessary to validate such methods (e.g. *Escherichia coli*, with the Ishii *et al*. 2007 dataset [27]). In multispecies modeling of microbial consortia, data on monoculture and community growth rates are much more abundant [21, 46–51] than data on monoculture internal fluxes, and data on co-culture internal fluxes are virtually nonexistent, as they require labor intensive methods like co-culture ^13^C-MFA [39]. The availability, or lack thereof, of some kinds of data will delimit the space of predictions that can be validated and predictive models that can be confidently presented. This inspires Q2.

Finally, successful predictive modeling in the field of omic integration is an iterative process, where the suitability of models and methods for particular applications can be traced back through the literature. For example, in Tian and Reed 2018 [24], the authors test their newly developed method using, among others, experimental data from a yeast aerobicity multi-omics dataset [28, 29] along with a yeast metabolic model from [52]. The metabolic model had, in its original publication, been validated on its ability to reproduce experimental observations related to aerobic and anaerobic conditions, giving the authors of [24] a solid foundation upon which to assess the model’s performance with their new method. Moreover, the datasets used to validate the method presented in Tian and Reed 2018 [24] include, but are not limited to, those used by [23] to demonstrate the poor predictive accuracy of older omic integration techniques. This allows the method in [24] not only to be evaluated in comparison to preceding methods and the baseline of pFBA, but also provides reassurance that the method’s predictive improvements generalize beyond the datasets used in [23]. This inclusion of novel datasets for validation extends to other omic integration methods published after [23], including [25, 26]. This prevents new models and methods from being “overfit” to a specific set of common validation datasets, generating good predictions for these datasets but failing to generalize beyond them.

**Figure 1:**
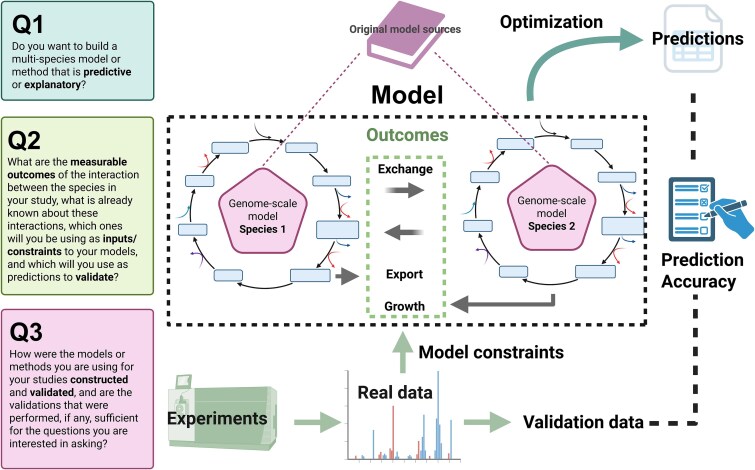
Visual representation of the guiding questions proposed for multispecies modeling studies in this review.

In the case of multispecies modeling, Joseph *et al.* [21] demonstrate that methods like MICOM, MMT, and COMETS, do a poor job of predicting co-culture growth ratios with both manually curated metabolic models and with automatically generated AGORA models. The latter, crucially, were not validated with respect to their relative growth rate predictions. And Scott *et al.* [22] demonstrate that, although the growth rates of their monoculture and co-cultured *C. kluyveri* [53] and *C. autoethanogenum* [54] models appear fairly accurate, the models do a poor job of predicting external production rates of target metabolites. This makes sense when we consider that attention was paid in the original studies that shared these models to growth rates, either through explicit validation or through an iterative model development process [53, 54]. Production of the target metabolites assessed in [22], on the other hand, was not validated at all in [54], and only qualitatively for two of the four metabolites in [53]. Q3 has been formulated from these observations. A visual reference for these three questions can be found in Fig. 1.

- Q1: Do you want to build a multispecies model or method that is predictive or explanatory?
- Q2: What are the measurable outcomes of the interaction between the species in your study, what is already known about these interactions, which ones will you be using as inputs/constraints to your models, and which will you use as predictions to validate? (It should be noted that different multispecies modeling methods also differ greatly in the number and nature of the parameters available to use as constraints.)
- Q3: How were the models or methods you are using for your studies constructed and validated, and are the validations that were performed (e.g. monoculture growth rates), if any, sufficient for predicting multispecies/community phenotypes?

## Case studies

### Optimization of bioreactors using multispecies modeling

Hanly, Urello, and Henson’s work on the optimization of *Saccharomyces cerevisiae* and *E. coli* co-cultures [3], which builds off their previous work on dynamic flux balance analysis (dFBA) modeling of *E. coli* strains [35], demonstrates how an iterative model-building and testing process with a strong focus on observing and optimizing the interaction between two species can result in highly predictive models.

The study follows an iterative workflow, with the authors’ goal being to optimize the performance of ethanol production from an *S. cerevisiae* and *E. coli* co-culture on mixed xylose and glucose feedstocks by determining initial *S. cerevisiae* and *E. coli* concentrations that result in simultaneous depletion of both substrates. The authors use experimental data on the biomass accumulation, substrate utilization, and ethanol production of *S. cerevisiae* and *E. coli* in batch culture individually to parameterize their dFBA models of these organisms, which were based on the preexisting reconstructions iND750 [55] and iJR904 [56]. Using these parameterized dFBA models, they then both modeled and measured substrate uptake, product formation, and biomass accumulation in a co-culture batch system. By examining the disagreement between their modeled and measured values, they were able to then parameterize the effects of interaction between their two species. Using this final version of the model, they were able to predict the initial inoculum concentrations for the two species that would result in simultaneous depletion of their feedstocks, which they then validated by setting up an experimental system with these predicted inoculum concentrations and confirming simultaneous depletion.

Referring to the list of questions to consider, this study is not only predictive, but it is built atop models that were intended to be predictive as well (Q1; Q3). In the original studies describing these models, the *S. cerevisiae* model was validated by showing good concordance between *in silico* knockout outcomes and empirical gene knockout datasets [55]. The *E. coli* model was not directly validated in this way, but is an iterative improvement on a previously published model, MG1655, which was [57]. The validation of *in silico* knockouts is fundamentally a way of interrogating the use of various metabolic subnetworks in an organism’s metabolic network under a given set of growth conditions [38]. So, to begin with, there is good evidence that the single species models being used have reasonably constructed metabolic networks.

However, these validations are not sufficient for Hanly, Urello, and Henson’s application, as they are primarily interested in substrate utilization, efficiency of biomass conversion, and ethanol production. Therefore, validations related to these quantities are essential. This was clearly recognized by the authors, because a great deal of effort was expended on measuring substrate utilization, biomass accumulation, and ethanol production and then parameterizing the models based on these measurements. In this case, the measurable outcomes of the cultured species’ interaction, substrate, biomass, and product levels in a bioreactor, were clearly defined, measurable, and used to successively parameterize and validate the models in mono- and co-culture (Q2). This is illustrative of how, if we clearly identify what characteristics of the models and/or workflows being used in a study were and were not validated, the lack of validation of input models and methods can be addressed through additional measurement and validation.

The authors also considered their prior knowledge of the *E. coli* and *S. cerevisiae* interaction (Q2). Since the environment in which the organisms interact is artificial and highly controlled, and the system is a synthetic consortium rather than an evolved interaction, the interaction between the organisms can be safely assumed to be purely at the level of exchanging metabolites via a shared external medium, without extensive metabolite cross-feeding. In this case, the extent to which the microorganisms can interact is determined strictly by their relative abilities to use external metabolites and their capacity, as determined by the transporters encoded in their genomes, to take up or export these metabolites.

The context of this Hanly *et al.* 2012 study [35] is arguably a best-case scenario, given that it features a single pairwise interaction between two species, features well-defined substrate inputs and all outputs of interest are measurable. Mapping the prediction process to Equation 1 in this case is simple: the input ***X*** is the starting concentrations of xylose and glucose, and the predicted outputs *E*(***Y***) are the substrate depletion time and the levels of xylose relative to the glucose at the end. The simplicity of the system allows for an iterative design process where previously unknown factors in the interspecies interaction, like the effect of ethanol on xylose uptake, can be discovered, added to the model, and then evaluated for their impact on predictive accuracy [35]. Many of these advantages may be absent outside the tightly controlled bioreactor context, and the study of naturally occurring microbial communities or symbioses may involve many more than two interacting partners.

### Modeling the microbial communities in cystic fibrosis sputum samples

In Henson *et al.* 2019 [7], the authors used a collection of AGORA [40] models together with the SteadyCom [8] framework to describe and predict the relative abundances of 17 bacteria representing the most abundant taxa in the lungs of individuals with cystic fibrosis (CF). The goal of this study was to understand how the readily measurable 16S rRNA abundances of microbes in CF patient airway samples could be used to predict the actual microbial community compositions and their interactions in these samples.

The authors compile a list of 17 common members of the CF airway microbial community from 16S rRNA data collected from a series of studies on CF patients [58–60]. They retrieved genome-scale models for these microbes from the AGORA database [40] and then parameterized an “average” nutrient composition of the airway environment by finding a nutrient composition that results in predicted species abundances in a consortium of commonly occurring microbial isolates in CF airways that mirror the averaged 16S rRNA data. The authors used this model in both a predictive and explanatory manner. Predictively, they were able to demonstrate that when specific monoculture pathogens are added to the consortium, the relative species abundances predicted by their parameterized consortium model aligned closely with the 16S rRNA estimated proportions measured for specific CF patients whose lungs were confirmed to harbor those pathogens (Q1). Explanatorily, the authors also demonstrated that the great variability seen in the CF airway communities of patients not harboring monoculture pathogens can be explained by natural variations in the nutrient composition of the lung environment. While this has not been tested, the explanatory modeling is being used here as a tool for hypothesis generation and no claims are being made as to whether or not it reflects reality.

This paper succeeds as a predictive modeling study despite its reliance on models sourced from the AGORA database and the SteadyCom framework, a combination that was shown in [22] to not generally succeed in predicting co-culture growth rates (Q3). Similar to the Hanly, Urello, and Henson paper [3], this study performs the validation and parameterization necessary, using the measurable data available to them, to ensure that the models and workflows that they are using are suitable for their purposes. The measurable data, in this case, consists of 16S rRNA taken from a diverse set of patient sputum samples (Q2). The models used for the study come from the AGORA database, which as previously discussed, received criticism for unrealistic growth rates [41]. However, the study features an evaluation of the growth rates of all the models grown in isolation. It shows that these single species growth rate values all lie in a reasonable range, mostly between ~0.1 and ~0.3 h^−1^. So, despite the original validations done on the AGORA database models perhaps being insufficient for many studies’ purposes, this secondary validation done in the study itself alleviates concerns about their use.

The authors also leverage some prior knowledge about these CF airway microbial communities to further refine their model. They cite previous work [61, 62] supporting the idea that microbes in bacterial communities actively cross-feed metabolites and they increased the upper bounds of exchanges of some central metabolites between members of the CF lung consortium accordingly.

This study uses the 16S rRNA data both to help in defining the nutrient environment, which in turn influences the growth rate results, as well as the validation of the modeling approach by comparing empirical and predicted species abundances. Typically using parts of the validation data to inform the model construction can be problematic [38], however, they also demonstrate that their model successfully predicts the species abundances in patient samples with rare pathogens, which the nutrient uptake rates were not tuned to match. This represents a more robust validation of their model that does not trivially follow from the fine-tuning procedure. Indeed, the same research group has gone on in future studies to validate other model predictions, including the inability of one common CF community species, *Prevotella melaninogenica*, to grow in monoculture despite its ability to grow in a consortium [63].

The previous two examples feature systems where two or more species in a microbial consortium interact primarily through the exchange of metabolites with a shared external medium and which do not exhibit strong symbiotic associations. What about cases where one or more species are obligate symbionts of another, with well-defined and highly evolved exchanges?

### Modeling the symbiosis of plants with N_2_ fixing bacteria

Several studies have been done on metabolic exchanges between microbial obligate symbionts of macroscopic eukaryotes and their hosts. Of particular note are a series of papers on multispecies interactions between microbial symbionts and insect hosts [4, 33, 34]. However, these studies are primarily impressive for their depth and hypothesis generation, rather than representing predictive models *per se*. For predictive modeling, the work that has been done on leguminous plants and their N_2_-fixing rhizobial partners stands out [5, 64]. We will focus on the analyses done in [5].

This study used existing metabolic models of the leguminous plant *Medicago truncatula* [64] and the N_2_ fixing rhizobium *Sinorhizobium meliloti* [65] to predict (i) the grams of carbon necessary for the plant to supply to the bacteroid to fix one gram of nitrogen, (ii) the nitrogen fixation rate, (iii) the growth rate of the plant, and (iv) the effect of gene deletions in the bacterial symbiont. Additionally, however, the model is also used as an exploratory tool for hypothesis generation and investigation of, for example, the preference of rhizobia symbionts for C4 dicarboxylates over sucrose as a carbon and energy source (Q1). Therefore, like the Henson *et al.* study, the model presented in [5] contains both predictive and explanatory elements.

To understand the success of the predictive modeling in this study, the input models, methods, and constraints must first be considered. The authors generate their predictions by interconnecting their *M. truncatula* and *S. meliloti* models with metabolite transporters based on prior literature and by representing the geometry of the root infection zone and rhizosphere in this symbiosis altogether in a model they call the “Virtual Nodule Environment,” or “ViNE.” The construction of the model is aided by the extensive characterization of the rhizobia–plant symbiosis in prior literature and the availability of measurable outcomes from this symbiosis (Q2). In contrast to the bioreactor setting, where co-cultured organisms formed naïve consortia with interactions only through simple exchange with the medium, or the lung microbiome setting, where a large number of organisms interact in ways that are difficult to empirically characterize, the legume/rhizobium symbiosis has been extensively studied from the perspective of species-to-species metabolic exchanges. This allows the authors to parameterize their model, via the imposition of constraints on the directionality of metabolite transport between the host and its symbionts, using prior information about the system. It also allows the authors to define and/or validate the model using measured relationships like the ratio of bacteroid biomass to nodule biomass (i.e. the specialized plant tissue that hosts the bacterium), the ratio of nodule biomass to total plant biomass, the ratio of exported carbon to imported fixed nitrogen in the plant, and the chemical forms of nitrogen and carbon exchanged between the two organisms. These extensive prior measurements of parameters are used by the authors to validate the predictiveness of their model.

Similar to the other two case studies, the authors perform additional validations on their input models and explicitly validate the predictions of their final ViNE model (Q3). The *M. truncatula* model was not subjected explicitly to any form of validation in its original study. The *S. meliloti* model, on the other hand, was subjected to extensive validation of its growth rate on different carbon sources, comparison with empirically measured central metabolic flux data, and growth/no-growth on a large number of carbon and nitrogen substrates. The final ViNE model is then validated with respect to the previously highlighted predictions, including overall plant growth rate.

The details of the biological systems being modeled, methods used, and data used for input and validation in this case-study and the two preceding it are summarized in Table 2.

**Table 2:** Summary of the details of the case studies reviewed in this manuscript.

	Case studies		
	**Hanly *et al.* 2012** [3]	**Henson *et al.* 2019** [7]	**Holland *et al.* 2023** [6]
**System**	*E. coli* + *S. cerevisiae* bioreactor	Human airway microbiota in CF patients	Plant–rhizobia symbiotic association
**Models**	*S. cerevisiae* genome-scale metabolic model [55] and *E. coli* genome-scale metabolic model [56]	Genome-scale models of individual microbial species, taken from the AGORA database [40], each representing a major taxonomic group present in CF patients’ airways	*Glycine max* genome-scale metabolic model [66] and *Bradyrhizobium diazoefficiens* genome-scale metabolic model [37]
**Methods**	dFBA with substrate uptake kinetics parameterized with experimental data	SteadyCom [8] with media nutrients tuned initially to reproduce observed relative abundances of community members	FBA in the bespoke Virtual Nodule Environment (ViNE) [5] framework
**Inputs**	Time course data of *E. coli* and *S. cerevisiae* biomass as well as substrate and ethanol concentrations in batch culture for uptake kinetic parameterization. Using these values, a preliminary simulation was performed, from which an additional interaction was parameterized	16S rRNA estimated abundances of bacterial genera, which were used to determine baseline nutrient uptake rates for the community	Constraints on known metabolite transports between host and symbiont, as well as constraints on plant and microbe biomass
**Validated outputs**	Initial *E. coli* and *S. cerevisiae* inoculum concentrations that result in simultaneous depletion of feedstock (xylose and glucose)	16S rRNA estimated community member proportions in the presence of specific microbial species	Host relative growth rate (RGR) and g C g^−1^ N (grams of carbon from host plant exchanged for grams of nitrogen from symbiont)

## Summary

Successful predictive modeling has been accomplished with both highly curated models and models reconstructed with semiautomated methods, like the AGORA models. A range of methods have been used to great effect depending on the specific system under consideration, from dFBA and methods incorporating community objectives to simpler static FBA. Despite the variety of approaches, several consistent features stand out in successful predictive multispecies modeling studies. First, they feature clearly stated information and/or assumptions about how the organisms in the system interact, which is then incorporated into the modeling framework. Second, the genome-scale models used are validated with respect to the quantities that are important for answering the questions the authors pose, either in the original studies that presented them or during the multispecies modeling process. Finally, empirical data describing some aspect of the multispecies interaction is used to validate initial predictions and sometimes to constrain the system or define key parameters. In many ways, these characteristics are what one would generally expect of a successful predictive modeling project. However, the key difference here is that empirical measurements concerning the individual species and the interaction between them are essential to a successful multispecies modeling study.

One take-home message from the multispecies modeling studies that have been done to date is that the outcomes of species interactions are rarely, if ever, a trivial consequence of the metabolic network architectures of the interacting species combined with a naïve assumption of maximized growth of the individual interacting partners. Rather, careful consideration of the nature of known or theorized interactions, relevant kinetics parameters, and observable outcomes of the interactions are necessary to be successful. This, together with challenges related to model curation, perhaps explains the difficulties that have been encountered in formulating workflows that can consistently predict the outcomes of interspecies interactions [21].

## Future directions

There is increasing interest in understanding and modifying multispecies interactions, from using synthetic microbial consortia for biochemical production [67], to the manipulation of the microbial communities associated with crops and with humans for agricultural and biomedical applications [68, 69]. Identifying what modifications to these complex systems may provide benefits will require predictive modeling techniques, making the development of this area important for future biotechnological applications. Currently available methods for predicting the outcomes of microbial interactions show poor predictive accuracy; however, as shown in the case studies above, specific examples of accurate predictive modeling in this area do exist. As more work is done that presents accurate predictive multispecies models, it is our hope that general principles will emerge that can then be translated into more robust and reliable algorithms for predicting interspecies interactions. Such efforts would benefit greatly from the generation of large datasets that report not only growth rate interactions between microbial species, but also metabolomic profiles.

As noted when discussing the findings of [41], the quality of the species-specific metabolic networks used to construct multispecies models can have a large impact on predictive accuracy. The development of more advanced methods for constructing metabolic networks, especially from complex metagenomic datasets [70], as well as the curation of databases of such models [42], could improve the predictive accuracy of studies going forward. However, benchmarking studies need to be performed to assess whether these new methods and databases genuinely improve predictive accuracy.

Another data type that would be extremely valuable, but that is currently quite scarce, is internal flux measurements. Except for the validation of *S. meliloti* in [65], none of the models or studies in question featured validation of flux predictions against internal flux measurements, which represents the gold standard when validating mono-species models [38]. It is possible to characterize exchanges between organisms in a more experimentally tractable way than performing a full ^13^C-MFA study by, for example, tracking the movement of radioisotopes or stable isotopes of carbon, nitrogen, or phosphorus between organisms in a multispecies system [71–74]. However, the level of detail provided by a full ^13^C-MFA analysis allows researchers to move from simple quantification of exchanges to detailed characterization of the resulting internal flux distributions in the exchanging partner species. Workflows have been developed to enable multispecies ^13^C-MFA [39] and have been deployed, to date, to understand exchanges in *E. coli* co-culture systems [75, 76]. Extension of ^13^C-MFA to other systems would provide valuable internal flux data both for multispecies model constraint and validation.

Key Points

- Predictive models describing multispecies metabolic interactions will be important as interest in biotechnological modifications of such interactions for industrial bioproduction, agriculture, and medicine grows.
- Current general-purpose algorithms for predicting multispecies metabolic interactions show poor performance, though individual models and studies show that successfully modeling such interactions is possible.
- The current state of the field mirrors that of a similar area—omic integration for metabolic flux prediction—roughly a decade ago.
- The emphasis on rigorous and standardized validation that helped the omic integration field move past its initial struggles should be applied to multispecies metabolic modeling as well.

## Data Availability

No data or code was generated as part of this review article.
